# Shape Covariation (or the Lack Thereof) Between Vertebrae and Other Skeletal Traits in Felids: The Whole is Not Always Greater than the Sum of Parts

**DOI:** 10.1007/s11692-017-9443-6

**Published:** 2018-01-10

**Authors:** Marcela Randau, Anjali Goswami

**Affiliations:** 1 Department of Genetics, Evolution and Environment, University College London, Darwin Building 218, Gower Street, London, WC1E 6BT UK; 2 Department of Earth Sciences, University College London, London, UK; 3 Department of Life Sciences, The Natural History Museum, London, SW7 5DB UK

**Keywords:** Modularity, Integration, Morphological evolution, Vertebral column, Felidae, Morphology

## Abstract

Within carnivorans, cats show comparatively little disparity in overall morphology, with species differing mainly in body size. However, detailed shape analyses of individual osteological structures, such as limbs or skulls, have shown that felids display significant morphological differences that correlate with their observed ecological and behavioural ranges. Recently, these shape analyses have been extended to the felid axial skeleton. Results demonstrate a functionally-partitioned vertebral column, with regions varying greatly in level of correlation between shape and ecology. Moreover, a clear distinction is evident between a phylogenetically-constrained neck region and a selection-responsive posterior spine. Here, we test whether this regionalisation of function reflected in vertebral column shape is also translated into varying levels of phenotypic integration between this structure and most other skeletal elements. We accomplish this comparison by performing pairwise tests of integration between vertebral and other osteological units, quantified with 3D geometric morphometric data and analysed both with and without phylogenetic correction. To our knowledge, this is the first study to test for integration across a comprehensive sample of whole-skeleton elements. Our results show that, prior to corrections, strong covariation is present between vertebrae across the vertebral column and all other elements, with the exception of the femur. However, most of these significant correlations disappear after correcting for phylogeny, which is a significant influence on cranial and limb morphology of felids and other carnivorans. Our results thus suggest that the vertebral column of cats displays relative independence from other skeletal elements and may represent several distinct evolutionary morphological modules.

**Electronic supplementary material:**

The online version of this article (10.1007/s11692-017-9443-6) contains supplementary material, which is available to authorized users.

## Introduction

The relationship between form and function has been shown to be present in a widespread range of organismal traits, with several examples of correlated changes in shape to promote adaptation to specific ecologies (e.g., Hutchinson 2012; Irschick 2002; Moon 1999; Ercoli et al. 2012; Gonyea 1978; Stayton 2006, 2008; Lauder 1995; McInnes et al. 2011). However, in a scenario where distinct organismal structures show covariation among themselves, independent adaptation of each structure to its optimal function may be hindered. Specifically, if selection drivers and/or directions are not the same in covarying traits, selection in one part may be obstructed by either opposing or stabilizing forces on other covarying elements. Alternatively, a degree of independence may arise that allows for some decoupling between structures, and further independent change may follow. However simplified, these are the concepts on which the fields of integration (i.e., the overall covariation of traits) and modularity (i.e., the relative autonomy of integrated structures, which are termed modules, from other structures) have been based (Olson and Miller 1958).

This form-function relationship has been particularly well explored in studies of carnivoran evolution, potentially due to the charismatic status of most species in this mammalian order and consequent improved levels of ecological knowledge, which facilitate these comparisons. Specifically, ecological and life history specialisations regarding a wide range of traits, from diet to locomotion to mating strategies (e.g., Fabre et al. 2013a, b; Antón et al. 2004; Bertram and Biewener 1990; Hudson et al. 2011; Holliday and Steppan 2004; Van Valkenburgh 2007; Antón and Galobart 1999; Cuff et al. 2016a, b; Gonyea 1978; Meachen-Samuels 2010; Randau et al. 2016b; Salesa et al. 2010; Jones and Goswami 2010; Doube et al. 2009; Zhang et al. 2012), have been shown to correlate with aspects of skeletal shape in living and fossil carnivorans. Within this order, the family of cat species (Felidae) shows little morphological disparity when only gross anatomy is considered, as most species differ mainly in body size and display a typical hypercarnivorous morphotype (Ewer 1973; MacDonald et al. 2010; Sunquist and Sunquist 2002; Van Valkenburgh 2007; Holliday and Steppan 2004). Rigorous shape analyses, however, have shown that cranial, dental and limb traits can successfully distinguish species that differ in ecology, particularly regarding either prey size or locomotor style (Dayan et al. 1990; Meachen-Samuels and Van Valkenburgh 2009a, b; Gonyea 1978; Meachen-Samuels 2012). Nevertheless, limb and cranial shapes across Felidae have also been shown to be highly correlated with phylogeny (Martín-Serra et al. 2014a; Walmsley et al. 2012; Meloro and O’Higgins 2011; Meloro and Slater 2012; Piras et al. 2013). Recent work has shown that these ecologically-driven shape changes, although mostly concentrated in the cranium and limbs, are also present in vertebral morphology, although to a smaller and more regionalised degree. Specifically, it is at the posterior end of the vertebral column (i.e., T10–L7 vertebrae) that vertebral shape correlates most significantly with either body mass, prey size choice (i.e., specialisation in small, mixed, and large prey), or locomotor mode (i.e., cursorial, terrestrial, scansorial, and arboreal) (as discussed in Randau et al. 2016a, b), whilst vertebrae in the neck region are more conservative in shape. Even at this T10–L7 region, the amount of vertebral shape variation across species is only explained by ecology to a relatively small degree (i.e., prey size and locomotor mode explained around 18 and 12% of the shape variance, respectively; Randau et al. 2016a). In comparison, previous studies of felids have demonstrated that when using measurements of the skull and limbs it was possible to correctly discriminate between species’ ecology at around 65 and 93% of the time, respectively (Meachen-Samuels and Van Valkenburgh 2009a, b).

Furthermore, vertebral shape may be largely developmentally constrained across all regions of the axial skeleton, which would prevent more extensive changes in response to selection (Asher et al. 2011; Buchholtz 2012; Buchholtz et al. 2014; Richardson and Chipman 2003; Losos 2011; Galis et al. 2014; Cullinane 2000). The mammalian vertebral column has been suggested to be under strong canalisation and developmental stability, which may explain its reduced variability with regards to vertebral count when compared to other vertebrate groups (Buchholtz 2012; Buchholtz et al. 2012; Müller et al. 2010; Narita and Kuratani 2005). Furthermore, we have demonstrated that a signal of developmental origin is present in most individual vertebral shape across adult felids, with most vertebrae possessing two internal modules of high shape covariation that are reflective of developmental origin (Randau and Goswami 2017b).

Taken together, the regionalised ecological signal in the vertebral column and the higher levels of shape adaptation in other skeletal elements raise the question of whether these ecologically-driven shape changes are correlated. Alternatively, differential influences on vertebral shape versus the rest of the skeleton may be reflected in the levels of integration and modularity among these elements. Here we test for shape covariation between presacral vertebrae and other skeletal elements, including the skull, girdles and limbs, in nine species of living cats in which the vertebral form and function relationship has already been explored (Randau et al. 2016a, b; Randau and Goswami 2017b). Specifically, we assess whether vertebrae covary with other osteological structures within complex systems (e.g., individual bones within the forelimb) and whether vertebrae within the ecologically-informative T10–L7 region show more frequent or higher correlations with other ecologically-informative skeletal elements. To perform this analysis, we use a powerful method developed specifically for assessing covariation among divergent datasets: the two-block Partial Least Squares (PLS) analysis (Bookstein et al. 2003; Rohlf and Corti 2000).

## Materials and Methods

Using an Immersion Microscribe G2X (Solution Technologies, Inc., Oella, Maryland), three-dimensional (hereafter, 3-D) landmarks were collected on 29 osteological elements throughout the skeleton of nine living felid species. Visits to seven international museums resulted in a dataset of 40 near-complete specimens spanning these nine species, as even large collections hold a relatively small number of complete skeletons. Specimen number per species ranged from two in *Panthera leo* to eight in *Panthera pardus* (Table S1). Due to the analytical power issues that may be generated when having a low ratio between specimen and landmark numbers (Mitteroecker and Gunz 2009; Adams et al. 2013; Collyer et al. 2015; Adams 2014; Cardini and Loy 2013), and the difficulty in obtaining large intraspecific sample sizes for complete skeletons of felids, the analyses shown here were performed across an interspecific dataset, with a phylogenetically informed framework (see below). Further, other analytical precautions were taken to ascertain the reliability of our results, including assessing the repeatability of the covariance matrices under resampling (Goswami and Polly 2010b; Melo et al. 2016), and comparing the significance of results to simulated samples of the same size, which were themselves generated by random permutations (i.e., non-parametric) of the original dataset (Adams and Collyer 2009; Collyer et al. 2015). The comprehensive element sampling of this analysis (i.e., spanning nearly the complete skeleton of the chosen specimens) is novel in morphological studies, and this broader approach offers new insights into shape evolution.

The skeletal elements included were: 19 presacral vertebrae (C1, C2, C4, C6, C7, T1, T2, T4, T6, T8, T10, T11, T12, T13, L1, L2, L4, L6, and L7), skull, dentary, scapula, forelimb long bones (i.e., humerus, radius and ulna), innominates, hindlimb long bones (i.e., femur and tibia), and sacrum. Axial and pelvic girdle elements (i.e., vertebrae, skull, dentary, sacrum, and innominates) were landmarked across the whole structure. All other bones were paired skeletal structures and were only landmarked on the left side of the skeleton (i.e., left scapula, humerus, radius, ulna, femur, and tibia). Due to the nature of museum specimens, most innominate specimens were separated into halves, and therefore the left and right sides had to be landmarked, and hence analysed, separately. Selection of vertebral types was done per the reasoning described in our previous studies (Randau et al. 2016a, b; Randau and Goswami 2017b, a). In summary, analyses including all vertebrae in the presacral column demonstrated that correlations between vertebral shape and ecological signal were heterogeneous throughout the vertebral column (Randau et al. a, 2016b), and that the gradual change in vertebral morphology within the traditional regions (i.e., cervical, thoracic and lumbar) would allow for subsampling of vertebral units, in exchange for expanded specimen sampling, without significant loss of biological information. Whereas this set assured thorough sampling of each region, it also included all vertebrae with distinct and unique morphology (e.g., C1 and C2), vertebrae which have been suggested to be biomechanically informative (e.g., the diaphragmatic T10 and the anticlinal T11), and vertebrae which were immediately placed at the boundaries between regions and the two vertebrae immediately before and after this pair (e.g., C7 and T1, and C6 and T2, respectively).

**Fig. 1:**
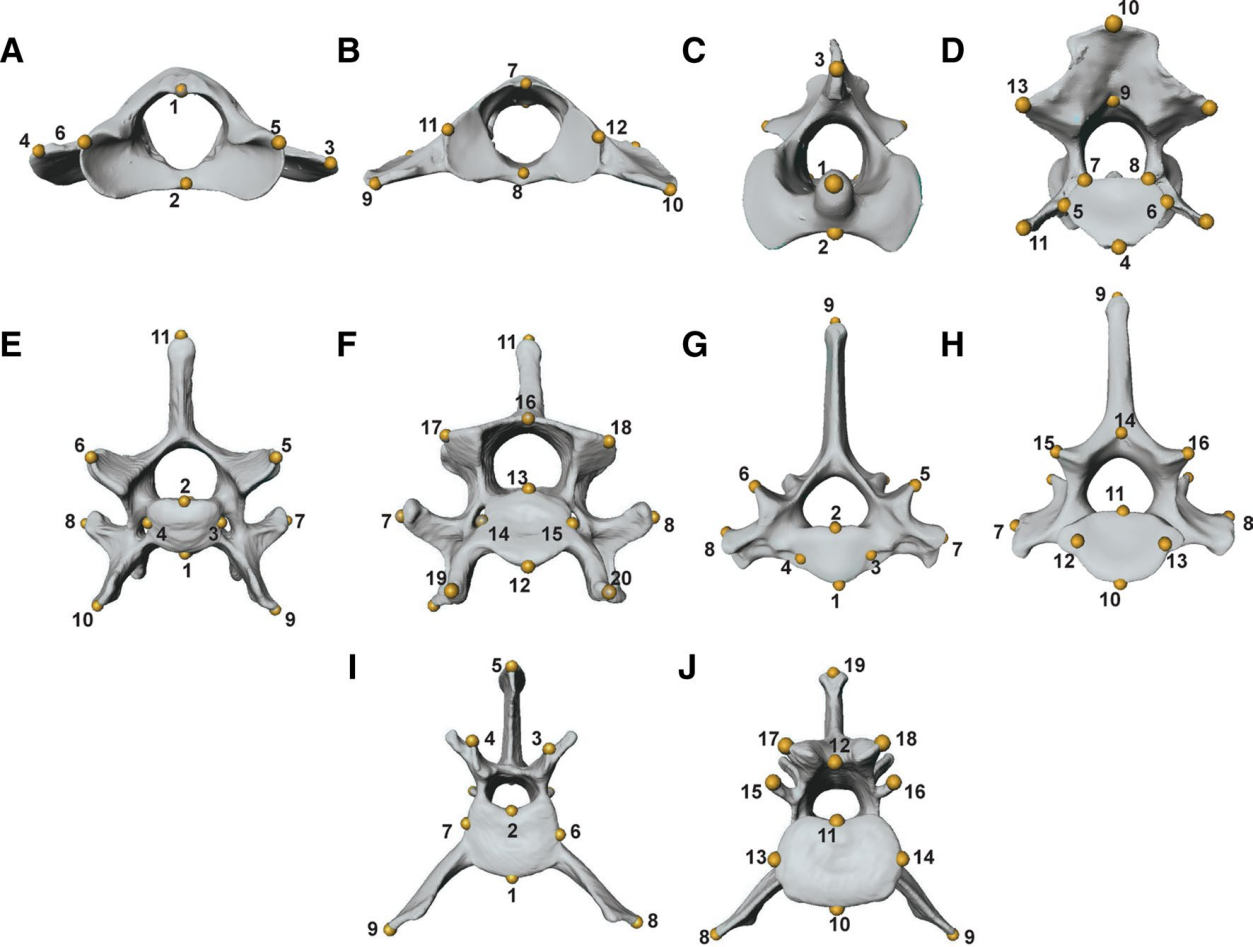
Three-dimensional models of presacral vertebrae of a cheetah (*Acinonyx jubatus*, USNM520539) illustrating their respective landmarks. Models represent the anterior and posterior views of: **a, b** atlas C1; **c, d** axis C2; **e, f** C6; **g, h** T1; and **i, j** L1. For the list of all landmarks and their description, see Table S2

**Fig. 2:**
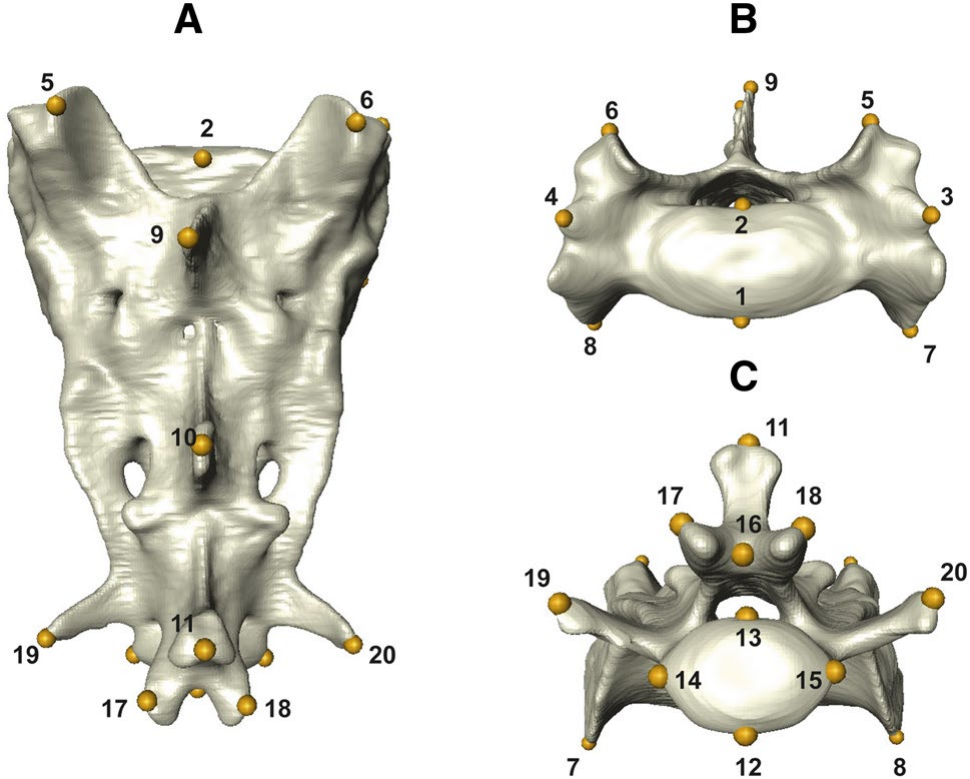
Three-dimensional model of the sacrum of a cheetah (*Acinonyx jubatus*, USNM520539) in dorsal (**a**), anterior (**b**), and posterior (**c**) views, showing position of three-dimension landmarks. Analysed landmarks were collected directly from osteological specimens. For the list of all landmarks and their description, see Table S2

**Fig. 3:**
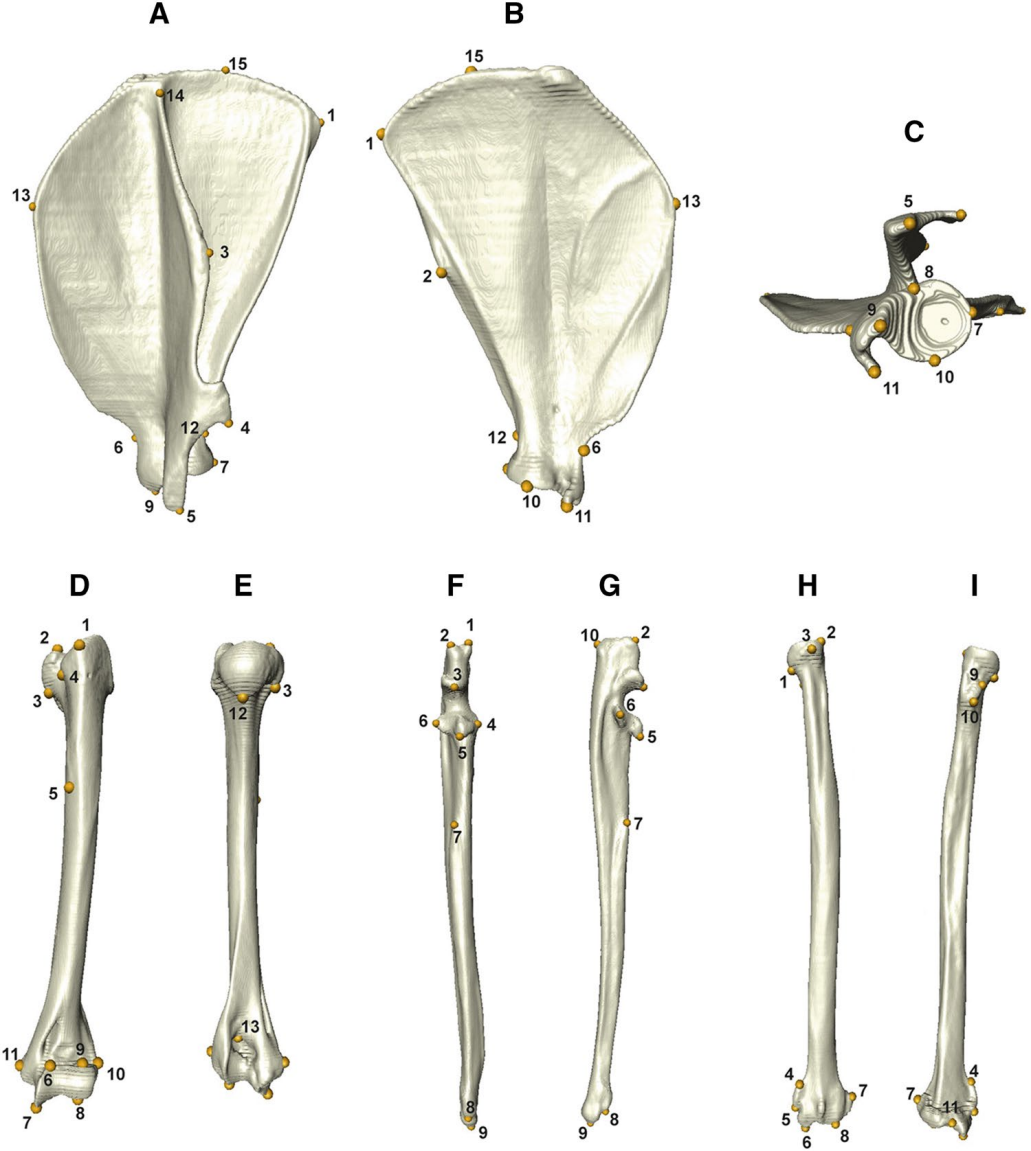
Three-dimensional model of the elements of the pectoral girdle (scapula) and forelimb with their respective landmarks. The scapula is shown in lateral (**a**), medial (**b**), and ventral (**c**) views. The humerus (**d** and **e**), the ulna (**f** and **g**) and the radius (**h** and **i**) are shown in anterior (**d, f** and **h**) and posterior (**e, g** and **i**) views. Elements are not to scale. The scapula and humerus represent elements of a serval (*Leptailurus serval*, NHM 133), while the ulna and radius are models of domestic cat (*Felis catus*, RVC21) bones. For the list of all landmarks and their description, see Table S2

**Fig. 4:**
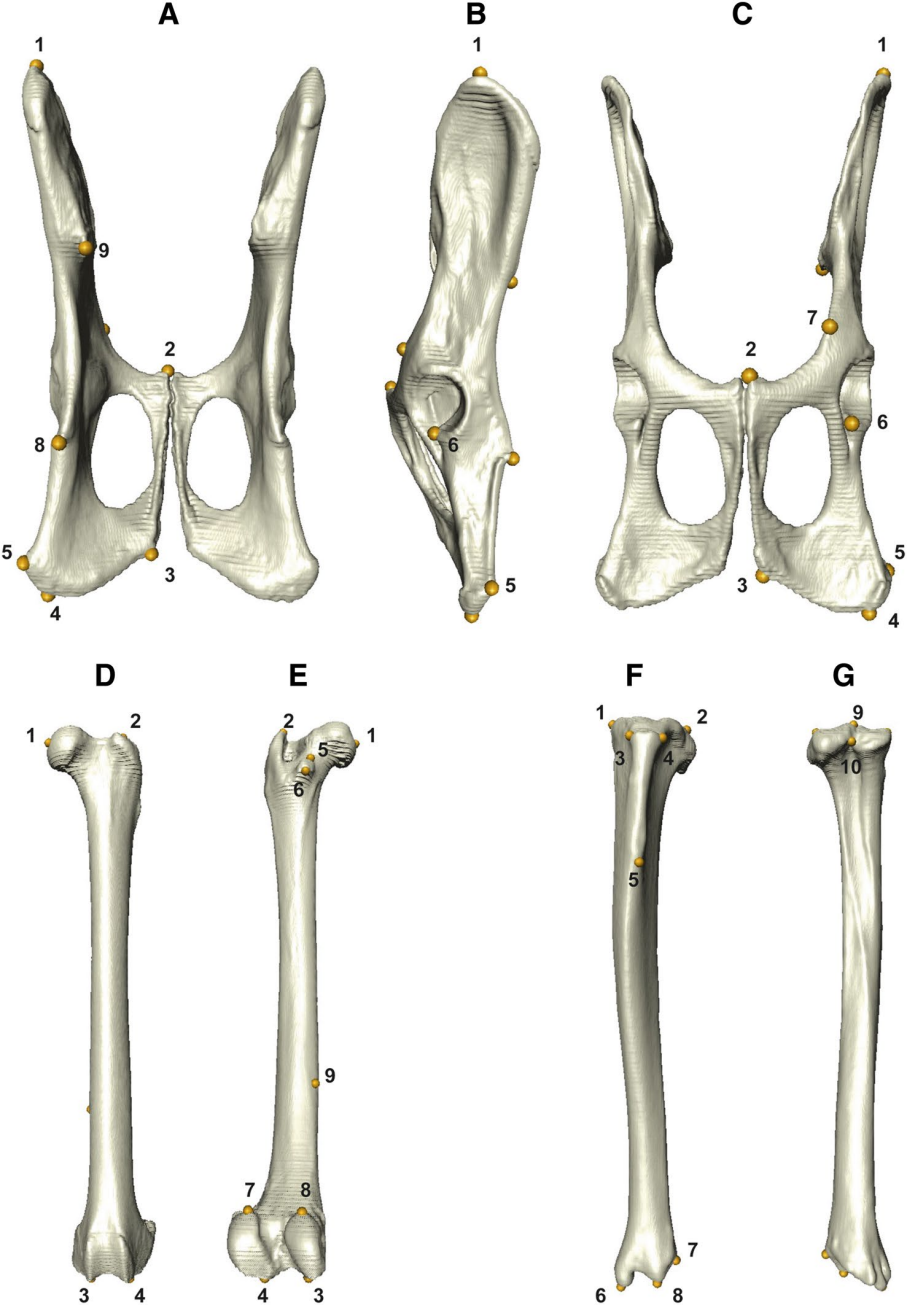
Three-dimensional model of the elements of the pelvic girdle (i.e., innominates), femur and tibia with their respective landmarks. The innominates are shown in dorsal (**a**), lateral (**b**), and ventral (**c**) views. Landmarks were taken on both innominates but here only shown on left side. The femur (**d** and **e**), and the tibia (**f** and **g**) are shown in anterior (**d** and **f**) and posterior (**e** and **g**) views. Elements are not to scale. The innominates and femur represent elements of a serval (*Leptailurus serval*, NHM 133), while the tibia belongs to a domestic cat (*Felis catus*, RVC21). For the list of all landmarks and their description, see Table S2

**Fig. 5:**
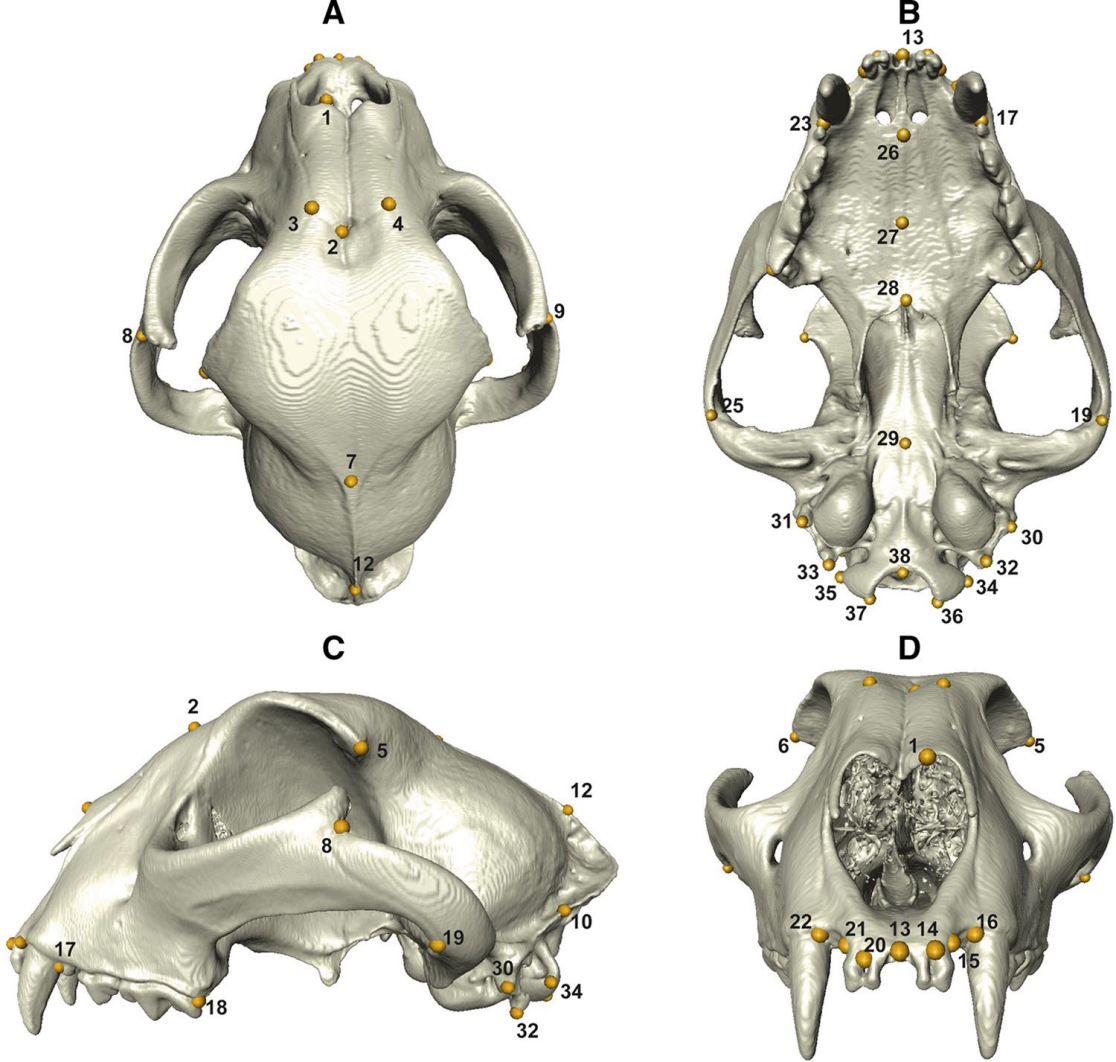
Three-dimensional model of the skull of a cheetah (*Acinonyx jubatus*, USNM520539) showing the three-dimensional landmarks that were collected in dorsal (**a**), ventral (**b**), lateral (**c**) and frontal (**d**) views. For the list of all landmarks and their description, see Table S2

**Fig. 6:**
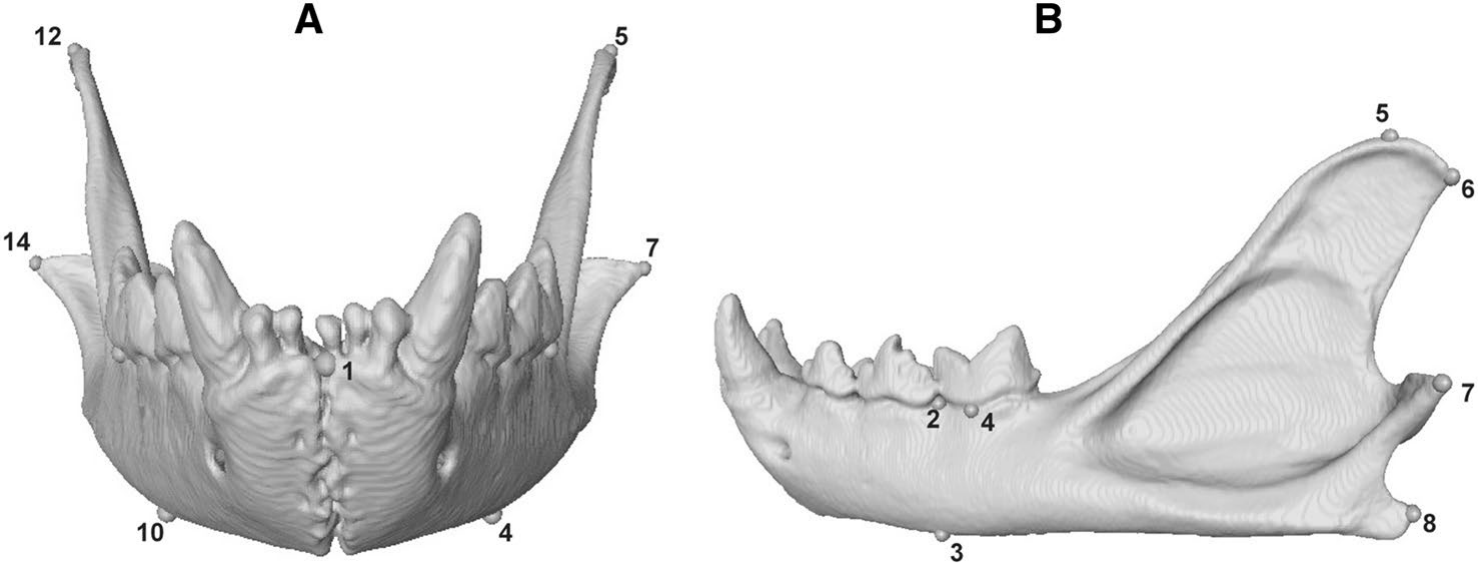
Three-dimensional model of the dentary of a cheetah (*Acinonyx jubatus*, USNM520539) showing the three-dimensional landmarks that were collected in frontal (**a**) and lateral (**b**) views. For the list of all landmarks and their description, see Table S2

Species analysed here included: cheetah (*Acinonyx jubatus*), puma (*Puma concolor*), lion (*Panthera leo*), leopard (*Panthera pardus*), clouded leopard (*Neofelis nebulosa*), serval (*Leptailurus serval*), leopard cat (*Prionailurus bengalensis*), ocelot (*Leopardus pardalis*), and domestic cat (*Felis catus*). These species represent the ranges of body mass and ecological (locomotory and prey size specialisations) spectra observed across the extant species of the Felidae family (Table 1, and Table S1 for specimen numbers), with examples of cursorial to arboreal felids that specialise in small, mixed and large species (MacDonald et al. 2010; Meachen-Samuels and Van Valkenburgh 2009a, b; Sunquist and Sunquist 2002). Landmark identities and numbers were object-specific, and varied from 12 (C1) to 17 (L6 and L7) in presacral vertebrae, and from nine (innominates, on each side) to 38 (skull) in all other elements (Table S2, and Figs. 1, 2, 3, 4, 5, 6 for landmarks’ positions).

**Table 1:** Felid species included in the studies and information on their ecological categories

Species	Common name	Prey size	Locomotion
*Acinonyx jubatus*	Cheetah	Large	Cursorial
*Felis catus*	Domestic cat	Small	Scansorial
*Leopardus pardalis*	Ocelot	Small	Arboreal
*Leptailurus serval*	Serval	Small	Terrestrial
*Neofelis nebulosa*	Clouded leopard	Mixed	Arboreal
*Panthera leo*	Lion	Large	Terrestrial
*Panthera pardus*	Leopard	Large	Scansorial
*Prionailurus bengalensis*	Leopard cat	Small	Scansorial
*Puma concolor*	Puma	Large	Scansorial

Ecological variables were collected from the literature (MacDonald et al. 2010; Meachen-Samuels and Van Valkenburgh 2009a, b; Sunquist and Sunquist 2002)

### Testing Matrix Repeatability

The stability of the covariance matrices for vertebral and non-vertebral units tested here was assessed with a bootstrap analysis of each dataset over 10,000 times and using a random skewers analysis to compare the covariance matrices of the original and resampled datasets (Goswami and Polly 2010b; Melo et al. 2016). Results demonstrated that covariance matrix repeatability was high, with values ranging from 0.91 to 0.96 for vertebral datasets, and from 0.90 to 0.96 for the non-vertebral elements, with a median and a mean of 0.94. These results thus demonstrate that our sampling was sufficient for accurately estimating the covariance matrices.

### Data Analyses

All analyses carried out here were performed in R version 3.3.1 (R Core Team 2016), using the ‘geomorph’ package (Adams and Otarola-Castillo 2013). Prior to all subsequent analyses, each skeletal component was individually aligned with a generalised Procrustes superimposition (GPA) in order to extract shape coordinates by removing the effects of rotation, scale and translation.

Covariation between each of the presacral vertebrae included here and the other skeletal components was measured pairwise with a two-block Partial Least Squares (hereafter, PLS) analysis, using the ‘integration.test’ function in ‘geomorph’. PLS analyses find two independent axes which represent the greatest covariation between the pair of blocks, and are the standard methodology for testing for integration between two structures, whether different regions of a single element or entirely separate elements. Importantly, because PLS analyses do not take into consideration the variation within each of the structures, this methodology is appropriate for testing integration between highly divergent structures with distinct levels of complexity and within-structure variation, or even between a set of landmark coordinates and a vector of categories concerning an ecological variable (Klingenberg 2013; Rohlf and Corti 2000; Goswami and Polly 2010b; Bookstein et al. 2003; Fabre et al. 2017; Adams 2016; Adams and Felice 2014; Álvarez et al. 2015; Bastir et al. 2005; Hautier et al. 2012).

With the ‘integration.test’ function the significance of each PLS test was calculated using randomised permutation tests that sample from the original dataset to simulate new populations of the same size. For each round of permutation, the new test statistic is compared to the value calculated using the original data. The number of resampling rounds in which the new test statistic was the same or higher than the original value is then divided by the total number of permutations (i.e., the p value of the test). Finally, it is this ratio that indicates the significance level of the analysis (Adams and Otarola-Castillo 2013; Collyer et al. 2015).

The PLS analyses performed here calculated the correlation coefficient as a measure of the covariation between each pairwise comparison, with significance level set at p values equal or under 0.05.

In order to account for relatedness among the felid species in our sample, skeletal integration was also quantified with a phylogenetic Partial Least Squares analysis under a model of Brownian motion evolution (Adams and Felice 2014), and using a recent phylogeny of felids (Piras et al. 2013), which was pruned to include only the nine species studied here (Fig. S1). Phylogenetic PLS analyses performed with the ‘phylo.integration’ function in ‘geomorph’ use a phylogenetic generalised least square (PGLS) approach (which has more appropriate Type I error and statistical power than using phylogenetic independent contrasts) to calculate the evolutionary covariance matrix (Adams and Felice 2014). Prior to phylogenetic PLS analysis, landmark data for each element was first separated into species sets (e.g., landmark data for skull specimens of ocelots) and aligned with a GPA. These species-specific Procrustes coordinates were then used to calculate the mean species shape per each bone, which was then analysed with the ‘phylo.integration’ function in ‘geomorph’. Significance level was again set at p values equal or less than 0.05.

### Multiple Comparisons Correction of the Significance Level

The analyses of integration performed here involved a large number of pairwise comparisons (i.e., 209 tests of integration between pairs of vertebra x other skeletal elements). In order to correct for an increased chance of false positives (i.e., finding a p value < 0.05 purely due to chance) due to this large number of comparisons, a Benjamini-Hochberg procedure (Benjamini and Hochberg 1995) was applied, with a false discovery rate at 0.05 (McDonald 2014). The Benjamini-Hochberg correction method uses a ranking technique to account for false positives. First, a false discovery rate (*Q*) is chosen (e.g., 0.05). Then, the original p values are ordered in an ascending manner (i.e., from smallest to largest) and ranked from *i* = 1 (lowest) to *m* = the total number of tests. Benjamini-Hochberg critical values are calculated as (*i*/*m*)*Q* for each of the original p values. Finally, the largest p value that is still lower than its assigned Benjamini-Hochberg critical value is determined as the significance threshold. P values that are equal to or lower than this new significance threshold are classified as significant (Benjamini and Hochberg 1995; McDonald 2014).

### Allometry

Shape coordinates for vertebral and other skeletal traits were not directly corrected for allometry prior to the integration analyses. Importantly, due to the high correlation of body size and evolutionary relatedness in Felidae, further correction after applying a phylogenetic PLS would likely introduce error (also, see below for discussion of comparison of results of general and phylogenetic PLS analyses).

## Results

### Skeletal Shape Covariation

Without considering the effects of phylogeny, 198 of the 209 pairwise comparisons between vertebrae and other skeletal elements were significant (p value < 0.05; Table 2 and S3). Ten of the 11 results that were not significant involved the femur and various vertebrae, and the eleventh non-significant result involved the C4 and the scapula. Across the significant results, 169 out of 198 showed high to very high integration (i.e., PLS correlations between 0.704 and 0.921) between vertebrae and the rest of the skeleton. Benjamini-Hochberg correction rendered only one additional result non-significant: the integration between L4 and the sacrum (Table 2).

**Table 2:** Results from the PLS analysis showing correlation levels in each pairwise comparison between vertebrae and other skeletal traits

	Skull	Dentary	Scapula	Humerus	Ulna	Radius	Sacrum	Inno. L	Inno. R	Femur	Tibia
Atlas	0.871	0.85	0.738	0.842	0.797	0.806	0.748	0.833	0.824	0.58*	0.855
Axis	0.913	0.888	0.818	0.891	0.776	0.839	0.864	0.917	0.919	0.55*	0.898
C4	0.855	0.8	0.643*	0.834	0.816	0.833	0.818	0.782	0.787	0.665	0.845
C6	0.853	0.811	0.733	0.877	0.801	0.859	0.847	0.85	0.856	0.722	0.857
C7	0.872	0.791	0.778	0.822	0.772	0.768	0.827	0.814	0.823	0.673	0.83
T1	0.835	0.758	0.744	0.752	0.719	0.738	0.782	0.801	0.815	0.688	0.791
T2	0.803	0.796	0.69	0.772	0.763	0.805	0.704	0.796	0.804	0.76	0.818
T4	0.783	0.831	0.738	0.827	0.683	0.765	0.751	0.781	0.787	0.514*	0.809
T6	0.772	0.811	0.749	0.849	0.773	0.856	0.78	0.729	0.715	0.529*	0.843
T8	0.722	0.762	0.678	0.769	0.751	0.776	0.768	0.736	0.726	0.5*	0.767
T10	0.727	0.696	0.684	0.833	0.773	0.822	0.697	0.716	0.668	0.433*	0.803
T11	0.787	0.712	0.67	0.838	0.78	0.783	0.72	0.695	0.655	0.77	0.844
T12	0.854	0.735	0.741	0.761	0.815	0.671	0.71	0.78	0.795	0.556*	0.795
T13	0.896	0.756	0.764	0.78	0.848	0.771	0.782	0.857	0.849	0.657	0.753
L1	0.851	0.716	0.732	0.681	0.781	0.689	0.75	0.885	0.863	0.515*	0.767
L2	0.884	0.732	0.783	0.798	0.825	0.734	0.76	0.921	0.892	0.538*	0.733
L4	0.869	0.711	0.793	0.68	0.817	0.791	0.647*	0.831	0.807	0.524*	0.673
L6	0.873	0.766	0.765	0.717	0.747	0.619	0.76	0.775	0.784	0.73	0.72
L7	0.797	0.645	0.684	0.566	0.575	0.543	0.697	0.767	0.779	0.611	0.543

Italics demark results which were not significant (p value > 0.05), and asterisk (*) marks the tests which were not significant after Benjamini-Hochberg correction. For brevity, the innominates have been abbreviated to ‘Inno.’, and the following letters ‘L’ and ‘R’ denote either the left or right side of this structure, respectively

### Phylogenetic Correction

In contrast to the uncorrected analyses, only 97 out of the 209 pairwise tests were significant when analysed with phylogenetic PLS (Table 3 and S4). As before, all of the significant results displayed very high correlations, with coefficients ranging between 0.829 and 0.985. However, correcting for multiple comparisons removed most of the significant results, and only 15 pairwise integration tests remained significant after correction (Table 3). Out of these 15 significant correlations, 11 involved vertebrae T10 to L2 versus four in the cervical region, while none was found involving the C7–T8 vertebrae.

**Table 3:** Results from the phylogenetic PLS analysis showing correlation levels in each pairwise comparison between vertebrae and other skeletal traits under a model of Brownian motion

	Skull	Dentary	Scapula	Humerus	Ulna	Radius	Sacrum	Inno. L	Inno. R	Femur	Tibia
Atlas	*0.903*	**0.941**	*0.876*	*0.859*	*0.846*	*0.859*	*0.887*	0.927	0.943	*0.744*	0.898
Axis	*0.901*	0.934	0.924	0.935	*0.881*	0.886	*0.863*	**0.965**	**0.979**	*0.766*	0.926
C4	*0.735*	0.918	0.952	*0.812*	0.916	*0.843*	*0.888*	*0.907*	0.919	*0.741*	*0.807*
C6	*0.941*	*0.923*	*0.954*	*0.93*	**0.985**	*0.901*	*0.961*	0.977	0.978	*0.94*	*0.94*
C7	0.963	0.915	0.935	0.915	**0.94**	*0.737*	*0.867*	0.946	0.94	0.929	0.91
T1	*0.831*	*0.851*	*0.875*	0.943	*0.83*	0.925	*0.827*	0.916	0.927	*0.807*	*0.838*
T2	*0.843*	0.91	*0.839*	*0.813*	*0.731*	*0.818*	*0.883*	*0.811*	*0.846*	0.915	*0.854*
T4	*0.695*	0.908	0.932	*0.836*	*0.866*	*0.845*	*0.761*	*0.854*	*0.873*	*0.678*	*0.814*
T6	*0.814*	0.947	0.92	0.945	*0.866*	0.912	*0.837*	*0.87*	*0.878*	*0.814*	0.929
T8	*0.931*	0.947	*0.87*	*0.873*	*0.895*	*0.896*	*0.892*	*0.919*	*0.927*	*0.832*	*0.874*
T10	*0.699*	*0.811*	0.891	**0.958**	*0.803*	0.898	*0.798*	0.932	0.917	0.928	0.863
T11	*0.681*	*0.826*	0.912	**0.968**	**0.943**	*0.832*	*0.89*	*0.757*	*0.717*	*0.646*	0.93
T12	*0.895*	0.888	0.93	*0.878*	0.92	*0.845*	*0.866*	0.914	0.909	*0.846*	**0.937**
T13	*0.902*	0.902	0.933	*0.879*	**0.964**	*0.752*	*0.859*	**0.966**	0.952	0.869	0.881
L1	*0.848*	0.896	0.937	0.888	**0.941**	*0.724*	*0.821*	**0.977**	**0.963**	*0.823*	*0.805*
L2	*0.857*	0.902	0.939	*0.852*	0.93	*0.695*	*0.799*	**0.983**	**0.969**	*0.788*	*0.805*
L4	*0.873*	0.901	0.943	0.894	0.934	*0.697*	*0.79*	0.935	0.926	0.829	*0.814*
L6	0.886	0.901	0.929	*0.869*	0.925	*0.671*	*0.805*	0.941	0.938	0.856	*0.851*
L7	0.955	0.939	0.939	*0.892*	0.939	*0.688*	*0.9*	0.964	0.952	0.935	*0.87*

Italics demarks results which were not significant (p value > 0.05), and bold formatting marks the tests which remained significant after Benjamini-Hochberg correction. Abbreviations ‘Inno. L’ and ‘Inno. R’ defined as above

## Discussion

Modularity is a prevailing characteristic of the vertebral column in felids (Randau et al. 2016a; Randau and Goswami 2017a, b), and most likely of mammals in general (Buchholtz 2007; Buchholtz et al. 2012). In fact, modular organisation is ubiquitous across multiple levels of structures in the skeleton of organisms, observed across functionally linked elements (e.g., modular organisation within entire limbs; Schmidt and Fischer 2009; Fabre et al. 2014; or across the vertebral column; Randau and Goswami 2017a) and within different components of individual elements (e.g., within the skull; Goswami 2006a; Goswami and Polly 2010a; within humeral shape; Arias-Martorell et al. 2014; or within vertebrae; Randau and Goswami 2017b). It may therefore be hypothesised that modularity is a universal characteristic of complex traits and may be expected to exist at even higher levels of organisation within organisms, such as between the vertebral column and the limbs or the skull.

**Fig. 7:**
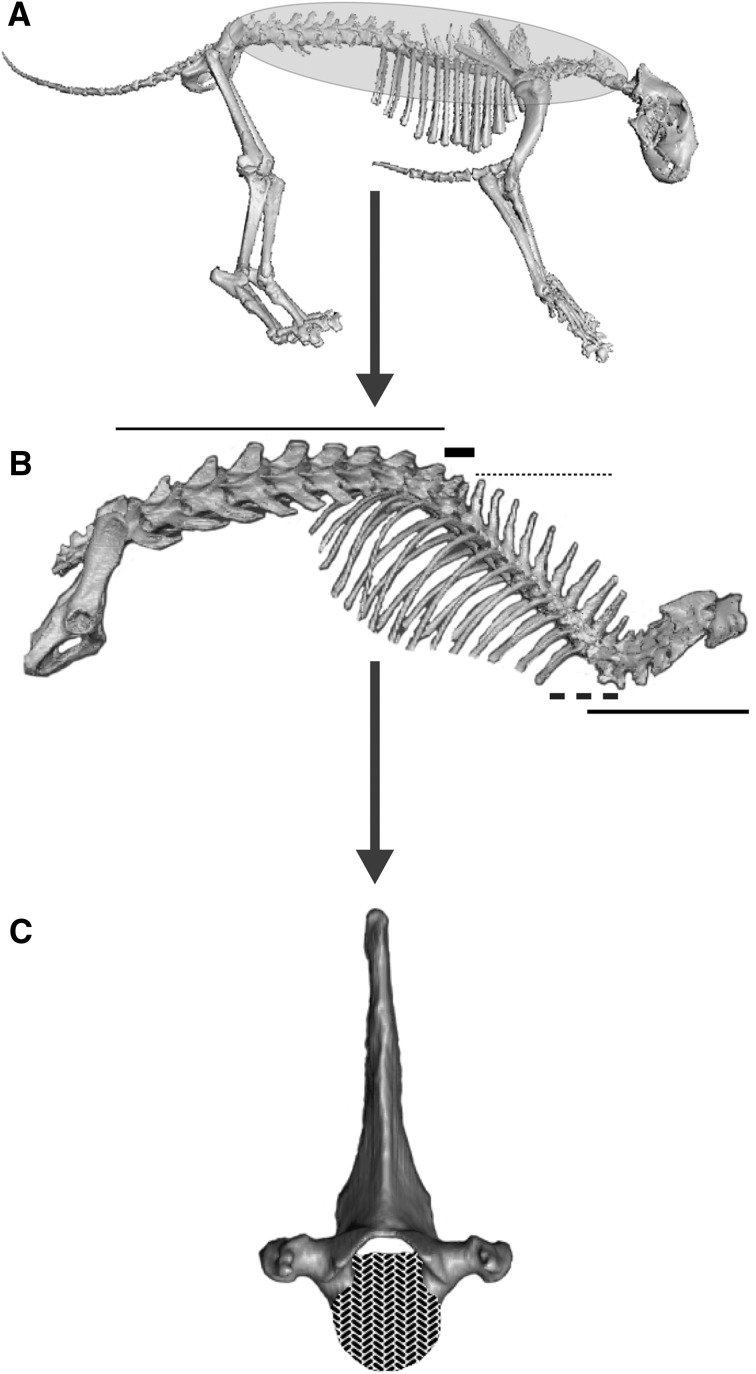
The hierarchical structure of modularity in the presacral vertebral column of felids. **a** The skeleton of a fossil American lion cheetah (*Panthera atrox*) showing the presacral vertebral column, marked in yellow and circled, as evolutionarily dissociated from the rest of the skeleton. **b** Within the vertebral column (here, a domestic cat specimen, *Felis catus*), five main intervertebral modules are suggested and coloured accordingly (represented by horizontal lines in printed b&w version): C1–C7 (in pink); T3–T9 (in yellow); an overlapping C6–T2 (in cyan), T10–T11 (in brown) and T12–L7 (in blue). **c** When the analysis is zoomed in to focus on individual vertebrae, most presacral vertebrae show shape covariation partitioned into two intravertebral modules, the centrum (in red, or highlighted with chevrons in printed b&w version) and the neural spine (in dark blue). Source: ‘A’ and ‘B’ were made using 3D reconstructions created and kindly supplied by Dr Andrew R. Cuff. (Color figure online)

Noticeably, as discussed previously (Randau and Goswami 2017a), the observed patterns of trait organisation are dependent on the level of analyses performed, as a hierarchical order has been demonstrated for the modular arrangement of biological traits: e.g., the mammalian skull has been demonstrated to be organised into multiple small partitions representing functional groups (Goswami 2006a; Goswami and Finarelli 2016; Cheverud 1995, 1982) that are defined within two larger blocks, each inclusive of a higher number of bones, that are observable when the focus of the analysis changes to a ‘face’ versus ‘neurocranium’ level (Drake and Klingenberg 2010). Similarly, a hierarchical organisation seems present in the presacral vertebral column of felids (Fig. 7) with the aforementioned two blocks within most individual vertebrae (Randau and Goswami 2017b), which are themselves partitioned between five larger modules across the spine, each including multiple vertebrae (Randau and Goswami 2017a). The results presented here add important detail to these organisational levels, strongly suggesting that the vertebral column, across all of its distinct modules, is evolutionary disassociated from other elements within the skeletal system of felids. This dissociation has the further consequence of suggesting that the previously identified morphological modules of the vertebral column are evolutionarily independent from proximal non-vertebral elements. Importantly, in light of the results shown here and in our previous work (Randau and Goswami 2017b, a), it becomes clear that these distinct levels of organisation are driven by either development or function, with each of these sources of covariation playing a more significant role in shape disparification (i.e., increase in variance) at different levels (e.g., the functional overprint of the developmental two-module model of intravertebral covariation discussed in Randau and Goswami 2017b). This heterogeneity in covariation patterns may reflect, or indeed allow biological organisation, and indicate both constraints (e.g., evolutionary history and development) and the product of selection (e.g., functional modules) (Wagner et al. 2007; West-Eberhard 2003; Wagner and Altenberg 1996; Raff 1996; Cullinane 2000; Rolian 2014; Porto et al. 2009; Nouailhetas Simon and Marroig 2017).

Studies of the vertebral column have shown that its function and organisation vary widely through time and across taxa. Large shifts in vertebral form and function have been observed in the shift from axial-driven to appendicular-focused locomotion, in the change to a parasagittal limb posture in mammals, and in the appearance of a muscularised diaphragm, which both affected locomotion and potentially constrained vertebral count (Schilling 2011; Buchholtz et al. 2012; Buchholtz and Stepien 2009). Additionally, the increase in regionalisation in the evolution of the mammalian axial skeleton has long been suggested to allow compartmentalisation of function across the vertebral series (Slijper 1946). Therefore, the mammalian vertebral column has been hypothesised to have experienced increases in complexity through time, even whilst being highly constrained throughout development (Buchholtz 2014, 2012).

This change in complexity and organisation in traits is central to the theory of modularity, by which higher independence between certain sets of traits may evolve to break constraints due to pleiotropy and canalisation, thus allowing further individual trait responses to selection (Goswami and Polly 2010a; Wagner 1996; Schlosser and Wagner 2004; Cheverud 1996). Further, whereas modularity may facilitate independent traits to undergo specific and more extensive changes, high levels of integration within modules or across overall structures have been suggested to also promote greater shape disparification if the main axis of variation agrees with the direction of selection (Goswami et al. 2014; Schluter 1996). This has been empirically observed in the vertebral column of felids, with vertebrae that have the highest levels of overall integration also displaying the greatest disparity (Randau and Goswami 2017b). On the other hand, integration across traits that are part of a functional unit is necessary to maintain coordination of shape changes across traits and preserve operative biomechanical systems, which means shape disparification of individual traits may be constrained by the integration across the system (Olson and Miller 1958). In carnivorans, high integration across functional units has been demonstrated in the forelimb of musteloids, with high covariation between bones forming and allowing the rotation of the lower arm (i.e., ulna and radius), and the bones forming the elbow joint (i.e., humerus and ulna, and ulna and radius), which is the key articulation allowing a plethora of behaviours (Fabre et al. 2014). Similarly, a recent study on the appendicular skeleton of terrestrial carnivorans (Martín-Serra et al. 2015) demonstrated that species that have a specialised cursorial mode of locomotion have higher covariation patterns across their limbs than non-cursorial taxa, and suggested that functional specialisation is correlated with an increase in integration.

Within the mammalian family of cats (Felidae), our recent work has shown a clear partitioning of the vertebral column into regions showing ecological specialisation and higher morphological disparity across species and regions with higher phylogenetic conservativeness (Randau et al. 2016a). We further identified a great degree of independence across these regions (Randau and Goswami 2017a). Specifically, ecology was shown to be correlated more strongly with vertebral shape in the posterior region (i.e., from the diaphragmatic T10 to the last lumbar L7), which also displayed the highest levels of intravertebral integration (Randau and Goswami 2017b), but not anteriorly (Randau et al. 2016a, b). These contrasting signals suggested a link between responsiveness to selection and a release from phylogenetic constraints or from functional constraints associated with the diaphragm and thus anterior to the T10–L7 axial region. This lack of uniformity in function was reflected in the sets of discrete morphological modules found across the vertebral column (Randau and Goswami 2017a), again corroborating with the hypothesis that increased modularity allows morphological change and adaptation to circumvent ancestral constraints.

Despite this significant ecological signal in the posterior vertebral column of felids, a comparative stronger ecological signal has been observed in other skeletal traits, such as the skull, mandible, and limbs (Dayan et al. 1990; Meachen-Samuels and Van Valkenburgh 2009a, b; Meachen-Samuels 2012; Meloro et al. 2013; Van Valkenburgh 2007; Samuels et al. 2013; Fabre et al. 2013b). This correlation between ecology and shape in other elements has, however, also been demonstrated to be highly dependent on phylogeny and body mass. After correcting for the influence of size and taxonomic relatedness on shape, the ecological signal across much of the skeleton in felids was usually largely reduced or removed (Martín-Serra et al. 2014a; Walmsley et al. 2012; Meloro and O’Higgins 2011; Meloro and Slater 2012; Piras et al. 2013). Body size has been suggested to be one of the main influences on musculo-skeletal shape in felids (Cuff et al. 2016a, b, 2015; Doube et al. 2009), but this trait too is heavily influenced by phylogenetic relationships among cats, with large species concentrated almost singularly in the genus *Panthera* (Johnson et al. 2006; MacDonald et al. 2010; Sunquist and Sunquist 2002; Cuff et al. 2015).

In this study, few correlations between the shapes of vertebrae and other skeletal traits were significant after correction for phylogeny and multiple comparisons. Among the results that were significant after all corrections, most (13 out of 15) involved forelimb elements (i.e., humerus and ulna) or the innominates. Although admittedly still in small numbers, most (11 out of 15) of these significant results involved vertebrae within the more ecologically disparate T10–L7 region, with the remaining four observed in the cervical region. Nevertheless, interpreting the functional signal of these results at the level of individual significant pairwise associations between vertebrae and elements of the forelimb and innominates are presently speculative without further development of the literature on vertebral biomechanics. Interestingly, however, results from both the analyses with and without phylogenetic correction showed little significant covariation between vertebral and femoral shapes. Although the femur was represented by relatively few landmarks, these results are unlikely to be due to a mere lack of shape characterisation, as the same or even smaller landmark numbers were used in other traits (ten in the ulna, and nine on each side of the innominates). However, these landmark numbers are comparable to or greater than the number of landmarks or measurements in other studies of limb integration and morphology (e.g., Meachen-Samuels and Van Valkenburgh 2009b; Martín-Serra et al. 2015, 2014a, 2014b; Walmsley et al. 2012; Samuels et al. 2013; Fabre et al. 2014). Moreover, a previous study reported increased effect of body size on femoral proportions in felids (Schmidt and Fischer 2009), which might contribute to its dissociation from vertebral morphology. However, this observation requires further study with a larger sample size in order to isolate other possible conflating factors. Generally, therefore, there is a consensus in the literature that both ecological signal and levels of integration across the appendicular and cranial skeletons of carnivorans are decreased or completely wiped out when phylogeny (or phylogenetically structured traits, such as body size) is taken into account (Martín-Serra et al. 2014b, 2015; Walmsley et al. 2012; Fabre et al. 2013a; Goswami 2006b).

The clear contrast between the strong influences of phylogeny and (strongly phylogenetically-structured) body mass on the shape of the cranium, limbs, and anterior vertebrae in felids (Randau et al. 2016a) may explain the large effect of phylogenetic correction on our results. Once phylogenetic effects are considered, the apparently strong shape covariation across the felid skeleton disappears almost entirely, suggesting that phylogeny, and with it body mass, may be the main forces shaping felid osteological morphology and skeletal integration in general.

Further, we previously identified strong integration within five vertebral modules across the presacral column, which were supported even after phylogenetic relationships were considered (Randau and Goswami 2017a). Taken together, the high integration within vertebral modules and the lack of correlation between those and other skeletal elements suggest that the vertebral column may be an independently evolving structure, relative to the other parts of the skeleton. These results suggest that, at the macroevolutionary scale, the vertebral column is not one evolving structure, but instead it is composed of independent morphological modules with distinct within-module constraints. Further, integration within these modules may be driven largely by different factors than that of other skeletal elements, specifically constrained by development as opposed to being responsive to ecology. Notably, the relatively widespread uniformity in presacral vertebral count across mammals, and even more so within Felidae (all cats present 27 presacral vertebrae), suggests that the mammalian presacral column is under strong developmental constraint (Asher et al. 2011; Buchholtz et al. 2012; Buchholtz and Stepien 2009; Fleming et al. 2015; Hautier et al. 2010; Müller et al. 2010; Narita and Kuratani 2005; Varela-Lasheras et al. 2011; Wellik 2007; Galis 1999; Cullinane 2000). In support of this hypothesis, we have previously confirmed that felid presacral vertebral shape is structured largely according to the developmental origins of vertebral components (i.e., ‘*centrum*’ versus ‘*neural-spine*’ related) (Randau and Goswami 2017b), demonstrating that development is also a strong constraint on changes in vertebral shape and not only in number. Although this conclusion may seem contradictory to the idea of diverse and regionalised vertebral shape in mammals evolving in response to meristic constrains (i.e., constraints on numbers), it may actually be the developmental signalling across the vertebrae that allows for greater shape disparity in areas of greatest integration (as observed in the T10–L7 region) (Randau and Goswami 2017b, a).

One of the limitations of this study was the restricted interspecific sample sizes. Due to the nature of large-vertebrate collections, it is not an easy feat to obtain large numbers of complete (or near-complete) specimens, but the results we present demonstrate the importance of comprehensively sampling the complete skeleton. We attempted to mitigate the unavoidable small sample sizes using multiple methods to both account for this issue and confirm the reliability of our results, the latter of which strongly indicates that our results are robust to the limitations of small sample sizes. Future work on model organisms may circumvent these issues, but this work will provide a useful template for macroevolutionary analyses spanning diverse, rare, or even extinct organisms.

Together, these observations support the inference that the lack of strong integration between the vertebral column and the rest of the skeleton is due to the different factors influencing the shape of each of these regions. Whilst studies of cranial and appendicular elements show that there is a strong correlation between shape and ecological specialisation, although this is strongly phylogenetically structured, developmental origin and processes may more highly influence and shape vertebral morphology.

## Electronic supplementary material

Below is the link to the electronic supplementary material.

Supplementary material 1 (DOCX 204 KB)
