# Supplementary material for: Shape Covariation (or the Lack Thereof) Between Vertebrae and Other Skeletal Traits in Felids: The Whole is Not Always Greater than the Sum of Parts
Source: Evol Biol. 2018 Jan 10;45(2):196–210. doi: 10.1007/s11692-017-9443-6 (PMC5938317; doi:10.1007/s11692-017-9443-6)

**Supplementary Information**

Table S1: Species and museum collection numbers for the specimens included in this study. Museum collection abbreviations are as follows: NHM: Natural History Museum, London; MNHN: Muséum National d’Histoire Naturelle, Paris; MCZ: Harvard Museum of Natural History, Cambridge; AMNH: American Museum of Natural History, New York; FMNH: Museum of Natural History, Chicago; USNM: Smithsonian National Museum of Natural History, Washington D.C.

| **Species** | **Specimen number** |
| --- | --- |
| *Acinonyx jubatus* | FMNH127834 |
|  | AMNH119655 |
|  | AMNH119656 |
|  | AMNH119657 |
| *Felis catus* | NHM 1952 10 20 4 |
|  | USNM 396268 |
|  | USNM 397631 |
| *Leptailurus serval* | FMNH 127843 |
|  | FMNH 44438 |
|  | FMNH 60491 |
|  | NHM 1845 9 25 23 |
| *Leopardus pardalis* | AMNH 214744 |
|  | AMNH 248728 |
|  | MNHN 1998 1866 |
|  | MNHN A3456 |
|  | USNM 271094 |
| *Neofelis nebulosa* | FMNH 54304 |
|  | MNHN 1961 217 |
|  | NHM 1965 1 18 1 |
|  | USNM 545387 |
| *Panthera leo* | AMNH 85147 |
|  | MCZ 9487 |
| *Panthera pardus* | AMNH 54462 |
|  | MNHN 1892 1079 |
|  | MNHN 1945 70 |
|  | MNHN A13045 1844 |
|  | MNHN A7932 |
|  | MNHN BII 4 |
|  | USNM 15684 |
|  | USNM 303320 |
| *Prionailurus bengalensis* | FMNH 99363 |
|  | NHM 1309b 1858 |
|  | NHM 1979 2895 |
|  | USNM 317283 |
| *Puma concolor* | AMNH10259 |
|  | AMNH90213 |
|  | FMNH129339 |
|  | MNHN1937 4 |
|  | USNM A21528 |
|  | USNM264166 |

Table S2: Landmark anatomical descriptions per vertebra and other skeletal traits.

| **Atlas** |  |
| --- | --- |
| 1 | Anterior mid-point of dorsal arch |
| 2 | Anterior mid-point of ventral arch |
| 3 | Anterior lateral-most tip of left transverse process |
| 4 | Anterior lateral-most tip of right transverse process |
| 5 | Dorso-anterior-most tip of left pre-zygapophysis |
| 6 | Dorso-anterior-most tip of right pre-zygapophysis |
| 7 | Posterior mid-point of dorsal arch |
| 8 | Posterior mid-point of ventral arch |
| 9 | Posterior lateral-most tip of left transverse process |
| 10 | Posterior lateral-most tip of right transverse process |
| 11 | Posterior-most tip of left post-zygapophysis |
| 12 | Posterior-most tip of right post-zygapophysis |
|  |  |
| **Axis** |  |
| 1 | Anterior-most point at tip of den |
| 2 | Ventral mid-point at base of den |
| 3 | Anterior-most point of neural spine |
| 4 | Posterior ventral mid-point of centrum |
| 5 | Posterior left lateral-most point of width of centrum |
| 6 | Posterior right lateral-most point of width of centrum |
| 7 | Posterior left dorso-lateral point of centrum |
| 8 | Posterior right dorso-lateral point of centrum |
| 9 | Posterior dorsal mid-point of the neural canal |
| 10 | Dorsal posterior-most point at tip of neural spine |
| 11 | Left lateral-most posterior tip of transverse process |
| 12 | Right lateral-most posterior tip of transverse process |
| 13 | Posterior-most dorsal point of left post-zygapophysis |
| 14 | Posterior-most dorsal point of right post-zygapophysis |
| 1 | Anterior ventral mid-point of centrum |
| 2 | Anterior dorsal mid-point of centrum |
| 3 | Anterior left lateral-most point of centrum |
| 4 | Anterior left lateral-most point of centrum |
| 5 | Anterior dorsal-most point of left pre-zygapophyses |
| 6 | Anterior dorsal-most point of right pre-zygapophyses |
| 7 | Anterior-most point of left lamina |
| 8 | Anterior-most point of right lamina |
| 9 | Dorsal-most point at tip of neural spine |
| 10 | Posterior ventral mid-point of centrum |
| 11 | Posterior dorsal mid-point of centrum |
| 12 | Posterior Left lateral-most point of centrum |
| 13 | Posterior right lateral-most point of centrum |
| 14 | Posterior dorsal mid-point of the neural canal |
| 15 | Posterior-most point of left post-zygapophyses |
| 16 | Posterior-most point of right post-zygapophyses |
| 17 | Lateral-most point of left transverse process |
| 18 | Lateral-most point of right transverse process |
|  |  |
| **C6** |  |
| 1 | Anterior ventral mid-point of centrum |
| 2 | Anterior dorsal mid-point of centrum |
| 3 | Anterior left lateral-most point of centrum |
| 4 | Anterior left lateral-most point of centrum |
| 5 | Anterior dorsal-most point of left pre-zygapophyses |
| 6 | Anterior dorsal-most point of right pre-zygapophyses |
| 7 | Lateral-most point of left transverse process |
| 8 | Lateral-most point of right transverse process |
| 9 | Anterior-most point of left lamina |
| 10 | Anterior-most point of right lamina |
| 11 | Dorsal-most point at tip of neural spine |
| 12 | Posterior ventral mid-point of centrum |
| 13 | Posterior dorsal mid-point of centrum |
| 14 | Posterior Left lateral-most point of centrum |
| 15 | Posterior right lateral-most point of centrum |
| 16 | Posterior dorsal mid-point of the neural canal |
| 17 | Posterior-most point of left post-zygapophyses |
| 18 | Posterior-most point of right post-zygapophyses |
| 19 | Posterior-most point of left lamina |
| 20 | Posterior-most point of right lamina |
|  |  |
| **C7 - T10** |  |
| 1 | Anterior ventral mid-point of centrum |
| 2 | Anterior dorsal mid-point of centrum |
| 3 | Anterior left lateral-most point of centrum |
| 4 | Anterior left lateral-most point of centrum |
| 5 | Anterior dorsal-most point of left pre-zygapophysis |
| 6 | Anterior dorsal-most point of right pre-zygapophysis |
| 7 | Lateral-most point of left transverse process |
| 8 | Lateral-most point of right transverse process |
| 9 | Dorsal-most point at tip of neural spine |
| 10 | Posterior ventral mid-point of centrum |
| 11 | Posterior dorsal mid-point of centrum |
| 12 | Posterior Left lateral-most point of centrum |
| 13 | Posterior right lateral-most point of centrum |
| 14 | Posterior dorsal mid-point of the neural canal |
| 15 | Posterior-most point of left post-zygapophysis |
| 16 | Posterior-most point of right post-zygapophysis |
|  |  |
| **T11** |  |
| 1 | Anterior ventral mid-point of centrum |
| 2 | Anterior dorsal mid-point of centrum |
| 3 | Anterior left lateral-most point of centrum |
| 4 | Anterior left lateral-most point of centrum |
| 5 | Anterior dorsal-most point of left pre-zygapophysis |
| 6 | Anterior dorsal-most point of right pre-zygapophysis |
| 7 | Posterior-most point of tip of left accessory process |
| 8 | Posterior-most point of tip of right accessory process |
| 9 | Dorsal-most point at tip of neural spine |
| 10 | Posterior ventral mid-point of centrum |
| 11 | Posterior dorsal mid-point of centrum |
| 12 | Posterior Left lateral-most point of centrum |
| 13 | Posterior right lateral-most point of centrum |
| 14 | Posterior dorsal mid-point of the neural canal |
| 15 | Posterior-most point of left post-zygapophysis |
| 16 | Posterior-most point of right post-zygapophysis |
|  |  |
| **T12 - T13** |  |
| 1 | Anterior ventral mid-point of centrum |
| 2 | Anterior dorsal mid-point of centrum |
| 3 | Anterior left lateral-most point of centrum |
| 4 | Anterior left lateral-most point of centrum |
| 5 | Anterior dorsal-most point of left pre-zygapophyses |
| 6 | Anterior dorsal-most point of right pre-zygapophyses |
| 7 | Anterior Dorsal-most point at tip of neural spine |
| 8 | Posterior Dorsal-most point at tip of neural spine |
| 9 | Posterior ventral mid-point of centrum |
| 10 | Posterior dorsal mid-point of centrum |
| 11 | Posterior Left lateral-most point of centrum |
| 12 | Posterior right lateral-most point of centrum |
| 13 | Posterior dorsal mid-point of the neural canal |
| 14 | Posterior-most point of left post-zygapophyses |
| 15 | Posterior-most point of right post-zygapophyses |
| 16 | Posterior-most point of tip of left accessory process |
| 17 | Posterior-most point of tip of right accessory process |
|  |  |
| **L1 - L4** |  |
| 1 | Anterior ventral mid-point of centrum |
| 2 | Anterior dorsal mid-point of centrum |
| 3 | Anterior dorsal-most point of left pre-zygapophyses |
| 4 | Anterior dorsal-most point of right pre-zygapophyses |
| 5 | Dorsal anterior-most point at tip of neural spine |
| 6 | Anterior left lateral-most point of centrum |
| 7 | Anterior left lateral-most point of centrum |
| 8 | Lateral-most point of left transverse process |
| 9 | Lateral-most point of right transverse process |
| 10 | Posterior ventral mid-point of centrum |
| 11 | Posterior dorsal mid-point of centrum |
| 12 | Posterior dorsal mid-point of the neural canal |
| 13 | Posterior Left lateral-most point of centrum |
| 14 | Posterior right lateral-most point of centrum |
| 15 | Posterior-most point of tip of left accessory process |
| 16 | Posterior-most point of tip of right accessory process |
| 17 | Posterior-most point of left post-zygapophyses |
| 18 | Posterior-most point of right post-zygapophyses |
| 19 | Dorsal posterior-most point at tip of neural spine |
|  |  |
| **L6 - L7** |  |
| 1 | Anterior ventral mid-point of centrum |
| 2 | Anterior dorsal mid-point of centrum |
| 3 | Anterior dorsal-most point of left pre-zygapophyses |
| 4 | Anterior dorsal-most point of right pre-zygapophyses |
| 5 | Dorsal anterior-most point at tip of neural spine |
| 6 | Anterior left lateral-most point of centrum |
| 7 | Anterior left lateral-most point of centrum |
| 8 | Lateral-most point of left transverse process |
| 9 | Lateral-most point of right transverse process |
| 10 | Posterior ventral mid-point of centrum |
| 11 | Posterior dorsal mid-point of centrum |
| 12 | Posterior dorsal mid-point of the neural canal |
| 13 | Posterior Left lateral-most point of centrum |
| 14 | Posterior right lateral-most point of centrum |
| 15 | Posterior-most point of left post-zygapophyses |
| 16 | Posterior-most point of right post-zygapophyses |
| 17 | Dorsal posterior-most point at tip of neural spine |
|  |  |
| **Sacrum** |  |
| 1 | Anterior ventral mid-point of centrum |
| 2 | Anterior dorsal mid-point of centrum |
| 3 | Anterior lateral-most point of left articular surface with pelvis |
| 4 | Anterior lateral-most point of right articular surface with pelvis |
| 5 | Anterior dorsal-most point of left pre-zygapophyses |
| 6 | Anterior dorsal-most point of right pre-zygapophyses |
| 7 | Anterior ventral-most point of left articular surface with pelvis |
| 8 | Anterior ventral-most point of right articular surface with pelvis |
| 9 | Dorsal-most point at tip of first neural spine |
| 10 | Dorsal-most point at tip of second neural spine |
| 11 | Dorsal-most point at tip of third neural spine |
| 12 | Posterior ventral mid-point of centrum |
| 13 | Posterior dorsal mid-point of centrum |
| 14 | Posterior Left lateral-most point of centrum |
| 15 | Posterior right lateral-most point of centrum |
| 16 | Posterior dorsal mid-point of the neural canal |
| 17 | Posterior-most point of left post-zygapophyses |
| 18 | Posterior-most point of right post-zygapophyses |
| 19 | Lateral-most point of left transverse process |
| 20 | Lateral-most point of right transverse process |
|  |  |
| **Scapula** |  |
| 1 | Most dorsal point of the posterior border |
| 2 | Ventral boundary of the teres major process |
| 3 | Posterior-most lateral mid-point of the scapular spine |
| 4 | Distal tip of the acromion process |
| 5 | Most posterior point of the metacromion |
| 6 | Point of maximum curvature at the posterior border of the neck |
| 7 | Most posterior point of the border of the glenoid fossa |
| 8 | Midpoint of the lateral border of the glenoid fossa (point of max curvature from lateral view) |
| 9 | Most proximal (anterior) point of the border of the glenoid fossa at the anterior side |
| 10 | Medial-most mid-point of the glenoid fossa |
| 11 | Medial-most tip point of the coracoid process |
| 12 | Point of maximum curvature at the anterior border of the neck |
| 13 | Anterior-most point of the anterior border |
| 14 | Dorsal-most point of the scapular spine |
| 15 | Dorsal-most mid-point of proximal facet |
|  |  |
| **Pelvis** | (Each side) |
| 1 | Anterior-most (maximum cranial) projection of the iliac wing |
| 2 | Anterior-most (maximum cranial) point of the pelvic symphysis |
| 3 | Posterior-most (maximum caudal) point of the pelvic symphysis |
| 4 | Ventral projection of the ischial tuberosity |
| 5 | Dorsal projection of the ischial tuberosity |
| 6 | Acetabular notch |
| 7 | Iliopectineal eminence |
| 8 | Dorsal-most point of spine of ischium |
| 9 | Posterior-most (maximum caudal) projection of the articular surface for sacrum |
|  |  |
| **Humerus** |  |
| 1 | Anterior-most point of the greater tuberosity |
| 2 | Anterior-most point of the lesser tuberosity |
| 3 | Proximal-most ventral point of the lesser tuberosity |
| 4 | Proximal-most point of the pectoral ridge |
| 5 | Junction point of pectoral ridge and deltoid ridge |
| 6 | Proximal-most point of the trochlea |
| 7 | Distal-most point of the trochlear ridge |
| 8 | Distal-most point on the capitulum |
| 9 | Proximal-most point of the capitulum at the anterior side |
| 10 | Lateral-most point of the lateral epicondyle |
| 11 | Medial-most point of the medial epicondyle |
| 12 | Distal-most point of the humeral head |
| 13 | Proximal-most point at the dorsal border of the olecranon fossa |
|  |  |
| **Ulna** |  |
| 1 | Proximo-anterior-most medial point of the edge of the olecranon process |
| 2 | Proximo-anterior-most lateral point of the edge of the olecranon process |
| 3 | Distal-most point of the superior edge of the trochear notch |
| 4 | Latero-distal-most point of the coronoid process in anterior view |
| 5 | Distal-most point of the radial notch in anterior view |
| 6 | Lateral-most point of the radial notch in anterior view |
| 7 | Antero-lateral tip of the interosseous crest of ulna |
| 8 | Distal-most point of articular facet with radius |
| 9 | Distal-most point of the tip of the styloid process |
| 10 | Proximo-posterior-most point of the edge of the olecranon process |
|  |  |
| **Radius** |  |
| 1 | Proximo-medial-most point of the head |
| 2 | Proximo-latero-most point of the fovea |
| 3 | Proximo-antero-most point of the fovea |
| 4 | Proximo-dorso-most point of the styloid process |
| 5 | Medial-most point of the styloid process |
| 6 | Distal-most point of the styloid process |
| 7 | Distal-most point of facet with ulna |
| 8 | Lateral-most mid-point of articular facet with ulna |
| 9 | Dorso-medial point of the bicipital tuberosity |
| 10 | Ventral point of the bicipital tuberosity |
| 11 | Proximo-dorsal point of articular surface |
|  |  |
| **Femur** |  |
| 1 | Proximo-medial-most point of the head |
| 2 | Proximo-most point of the greater trochanter |
| 3 | Distal-most medial edge point of the patellar trochlear |
| 4 | Distal-most lateral edge point of the patellar trochlear |
| 5 | Medial projection of the proximal diaphysis |
| 6 | Lateral projection of the proximal diaphysis |
| 7 | Proximal-most point on superior part of the lateral condyle |
| 8 | Proximal-most point on superior part of the medial condyle |
| 9 | Distal-most point of the linea aspera |
|  |  |
| **Tibia** |  |
| 1 | Proximal-most point on the medial edge of the medial condyle |
| 2 | Proximal-most point on the lateral edge of the lateral condyle |
| 3 | Proximal-most medial end of the tibial tuberosity |
| 4 | Proximal-most lateral end of the tibial tuberosity |
| 5 | Distal-most end of the margo cranialis (crista tibiae) |
| 6 | Distal-most tip of the medial malleolus |
| 7 | Lateral-most point of the fibula facet |
| 8 | Distal-most point of the trochlea tali |
| 9 | Proximal-most point in the intercondyloid area |
| 10 | Distal-most point of the intercondyloid area/Proximal-most point of the popliteal notch |
|  |  |
| **Skull** |  |
| 1 | Anterior-most left tip of nasal |
| 2 | Anterior mid-point (left) of frontal |
| 3 | Left suture between nasal-maxilla-frontal |
| 4 | Right suture between nasal-maxilla-frontal |
| 5 | Lateral-most point of left post-orbital process |
| 6 | Lateral-most point of right post-orbital process |
| 7 | Middle point of suture between frontal and parietal bones |
| 8 | Left lateral superior-most point of suture between zygomatic and squamosal |
| 9 | Right lateral superior-most point of suture between zygomatic and squamosal |
| 10 | Left lateral/superior-most suture between parietal/occipital/squamosal |
| 11 | Right lateral/superior-most suture between parietal/occipital/squamosal |
| 12 | Middle point of suture between parietal and interparietal bones |
| 13 | Anterior-most left tip of premaxilla |
| 14 | Anterior-most point of alveolar length of left I3 |
| 15 | Posterior-most point of alveolar length left of I3 |
| 16 | Anterior-most point of alveolar length of left Upper C |
| 17 | Posterior-most point of alveolar length of left of Upper C |
| 18 | Latero-posterior-most point of end of left P4’s alveolus |
| 19 | Posterior-most point of left zygomatic |
| 20 | Anterior-most point of alveolar length of right I3 |
| 21 | Posterior-most point of alveolar length right of I3 |
| 22 | Anterior-most point of alveolar length of right Upper C |
| 23 | Posterior-most point of alveolar length of right of Upper C |
| 24 | Latero-posterior-most point of end of right P4’s alveolus |
| 25 | Posterior-most point of right zygomatic |
| 26 | Anterior-mid point of maxilla suture |
| 27 | Posterior-mid point of maxilla suture |
| 28 | Posterior-mid point of palatine suture |
| 29 | Posterior-mid point of presphenoid |
| 30 | Left lateral-most ventral point of the mastoid process |
| 31 | Right lateral-most ventral point of the mastoid process |
| 32 | Posterior-most point of left jugular process |
| 33 | Posterior-most point of right jugular process |
| 34 | Left lateral-most point of occipital condylar breath |
| 35 | Right lateral-most point of occipital condylar breath |
| 36 | Left posterior-most point of occipital condyle forming border of foramen magnum |
| 37 | Right posterior-most point of occipital condyle forming border of foramen magnum |
| 38 | Posterior-most middle point of basioccipital (ventral limit of foramen) |
|  |  |
| **Dentary** |  |
| 1 | Dorsal-most anterior middle point |
| 2 | Dorsal-most point of left jaw depth at p4/m1 junction |
| 3 | Ventral-most point of left jaw depth at p4/m1 junction |
| 4 | Lateral middle point of left m1 alveolus (line from between paraconid and protoconid) |
| 5 | Dorsal-most tip of left coronoid process (lateral view) |
| 6 | Posterior-most point of left coronoid process |
| 7 | Lateral-most tip of left condyloid process |
| 8 | Dorso-posterior-most tip of left angular process |
| 9 | Dorsal-most point of right jaw depth at p4/m1 junction (Middle point between p4-m1) |
| 10 | Ventral-most point of right jaw depth at p4/m1 junction |
| 11 | Lateral middle point of right m1 alveolus (line from between paraconid and protoconid) |
| 12 | Dorsal-most tip of right coronoid process (lateral view) |
| 13 | Posterior-most point of right coronoid process |
| 14 | Lateral-most tip of right condyloid process |
| 15 | Dorso-posterior-most tip of right angular process |

Table S3: P-values from the PLS analysis showing correlation levels in each pairwise comparison between vertebrae and other skeletal traits. Italics demarks results which were not significant (p-value > 0.05), and asterisk (*) marks the tests which were not significant after Benjamini-Hochberg correction. Abbreviations ‘Inno. L’ and ‘Inno. R’ defined as above.

|  | **SKULL** | **DENTARY** | **SCAPULA** | **HUMERUS** | **ULNA** | **RADIUS** | **SACRUM** | **INNO. L** | **INNO. R** | **FEMUR** | **TIBIA** |
| --- | --- | --- | --- | --- | --- | --- | --- | --- | --- | --- | --- |
| **ATLAS** | 0.001 | 0.001 | 0.001 | 0.001 | 0.001 | 0.001 | 0.002 | 0.001 | 0.001 | *0.069** | 0.001 |
| **AXIS** | 0.001 | 0.001 | 0.001 | 0.001 | 0.001 | 0.001 | 0.001 | 0.001 | 0.001 | *0.129** | 0.001 |
| **C4** | 0.001 | 0.001 | *0.067** | 0.001 | 0.001 | 0.001 | 0.001 | 0.002 | 0.002 | 0.003 | 0.001 |
| **C6** | 0.001 | 0.001 | 0.019 | 0.001 | 0.001 | 0.001 | 0.001 | 0.001 | 0.001 | 0.003 | 0.001 |
| **C7** | 0.001 | 0.001 | 0.001 | 0.001 | 0.001 | 0.001 | 0.001 | 0.001 | 0.001 | 0.003 | 0.001 |
| **T1** | 0.001 | 0.001 | 0.005 | 0.001 | 0.001 | 0.002 | 0.002 | 0.001 | 0.001 | 0.004 | 0.001 |
| **T2** | 0.001 | 0.001 | 0.012 | 0.001 | 0.001 | 0.001 | 0.024 | 0.001 | 0.001 | 0.001 | 0.001 |
| **T4** | 0.001 | 0.001 | 0.003 | 0.001 | 0.006 | 0.001 | 0.002 | 0.001 | 0.001 | *0.333** | 0.001 |
| **T6** | 0.001 | 0.001 | 0.002 | 0.001 | 0.001 | 0.001 | 0.002 | 0.001 | 0.001 | *0.172** | 0.001 |
| **T8** | 0.003 | 0.001 | 0.036 | 0.001 | 0.001 | 0.001 | 0.005 | 0.001 | 0.001 | *0.392** | 0.001 |
| **T10** | 0.001 | 0.001 | 0.001 | 0.001 | 0.001 | 0.001 | 0.002 | 0.001 | 0.002 | *0.139** | 0.001 |
| **T11** | 0.001 | 0.001 | 0.002 | 0.001 | 0.001 | 0.001 | 0.003 | 0.002 | 0.005 | 0.001 | 0.001 |
| **T12** | 0.001 | 0.002 | 0.005 | 0.001 | 0.001 | 0.004 | 0.01 | 0.001 | 0.001 | *0.121** | 0.001 |
| **T13** | 0.001 | 0.001 | 0.001 | 0.001 | 0.001 | 0.001 | 0.001 | 0.001 | 0.001 | 0.011 | 0.001 |
| **L1** | 0.001 | 0.001 | 0.001 | 0.001 | 0.001 | 0.001 | 0.001 | 0.001 | 0.001 | *0.08** | 0.001 |
| **L2** | 0.001 | 0.001 | 0.001 | 0.001 | 0.001 | 0.001 | 0.001 | 0.001 | 0.001 | *0.062** | 0.001 |
| **L4** | 0.001 | 0.001 | 0.001 | 0.002 | 0.001 | 0.001 | 0.05* | 0.001 | 0.001 | *0.087** | 0.002 |
| **L6** | 0.001 | 0.001 | 0.001 | 0.001 | 0.001 | 0.005 | 0.003 | 0.001 | 0.001 | 0.001 | 0.001 |
| **L7** | 0.001 | 0.002 | 0.001 | 0.028 | 0.01 | 0.017 | 0.009 | 0.001 | 0.001 | 0.001 | 0.021 |

Table S4: P-values from the phylogenetic PLS analysis showing correlation levels in each pairwise comparison between vertebrae and other skeletal traits under a model of Brownian motion. Italics demark results which were not significant (p-value > 0.05), and bold formatting marks the tests which remained significant after Benjamini-Hochberg correction. Abbreviations ‘Inno. L’ and ‘Inno. R’ defined as above.

|  | **SKULL** | **DENTARY** | **SCAPULA** | **HUMERUS** | **ULNA** | **RADIUS** | **SACRUM** | **INNO. L** | **INNO. R** | **FEMUR** | **TIBIA** |
| --- | --- | --- | --- | --- | --- | --- | --- | --- | --- | --- | --- |
| **ATLAS** | *0.08* | **0.005** | *0.137* | *0.138* | *0.104* | *0.07* | *0.27* | 0.019 | 0.01 | *0.382* | 0.043 |
| **AXIS** | *0.087* | 0.013 | 0.049 | 0.016 | *0.078* | 0.036 | *0.461* | **0.001** | **0.001** | *0.357* | 0.008 |
| **C4** | *0.76* | 0.028 | 0.012 | *0.345* | 0.03 | *0.103* | *0.219* | *0.055* | 0.036 | *0.444* | *0.297* |
| **C6** | *0.258* | *0.239* | *0.154* | *0.19* | **0.002** | *0.182* | *0.178* | 0.009 | 0.008 | *0.092* | *0.106* |
| **C7** | 0.01 | 0.022 | 0.03 | 0.041 | 0.016 | *0.366* | *0.384* | 0.005 | 0.016 | 0.018 | 0.02 |
| **T1** | *0.433* | *0.211* | *0.204* | 0.02 | *0.229* | 0.017 | *0.664* | 0.034 | 0.022 | *0.313* | *0.276* |
| **T2** | *0.278* | 0.028 | *0.315* | *0.342* | *0.494* | *0.162* | *0.33* | *0.284* | *0.156* | 0.017 | *0.131* |
| **T4** | *0.79* | *0.023* | 0.022 | *0.172* | *0.071* | *0.076* | *0.858* | *0.107* | *0.08* | *0.517* | *0.134* |
| **T6** | *0.445* | 0.004 | 0.045 | 0.007 | *0.069* | 0.017 | *0.667* | *0.092* | *0.091* | *0.159* | 0.012 |
| **T8** | *0.084* | 0.028 | *0.352* | *0.248* | *0.097* | *0.054* | *0.503* | *0.063* | *0.05* | *0.252* | *0.187* |
| **T10** | *0.476* | *0.093* | 0.047 | **0.003** | *0.081* | 0.007 | *0.501* | 0.008 | 0.017 | 0.007 | 0.015 |
| **T11** | *0.56* | *0.099* | 0.022 | **0.002** | **0.002** | *0.052* | *0.14* | *0.238* | *0.437* | *0.401* | 0.009 |
| **T12** | *0.06* | 0.038 | 0.02 | *0.066* | 0.021 | *0.076* | *0.284* | 0.022 | 0.028 | *0.083* | **0.002** |
| **T13** | *0.053* | 0.023 | 0.03 | *0.064* | **0.003** | *0.236* | *0.331* | **0.002** | 0.008 | 0.037 | 0.039 |
| **L1** | *0.111* | 0.023 | 0.021 | 0.044 | **0.002** | *0.143* | *0.417* | **0.001** | **0.002** | *0.052* | *0.091* |
| **L2** | *0.074* | 0.016 | 0.022 | *0.065* | 0.009 | *0.202* | *0.498* | **0.001** | **0.001** | *0.076* | *0.074* |
| **L4** | *0.06* | 0.009 | 0.02 | 0.047 | 0.013 | *0.18* | *0.535* | 0.007 | 0.017 | 0.045 | *0.069* |
| **L6** | 0.044 | 0.015 | 0.031 | *0.064* | 0.019 | *0.415* | *0.535* | 0.01 | 0.015 | 0.048 | *0.056* |
| **L7** | 0.015 | 0.006 | 0.035 | *0.103* | 0.027 | *0.597* | *0.355* | 0.01 | 0.011 | 0.015 | *0.082* |

Fig S1: Phylogenetic relationships among the species included in the analyses. The displayed topology was based on a recent phylogeny of felids (Piras et al. 2013), which was pruned to include only the nine species studied.


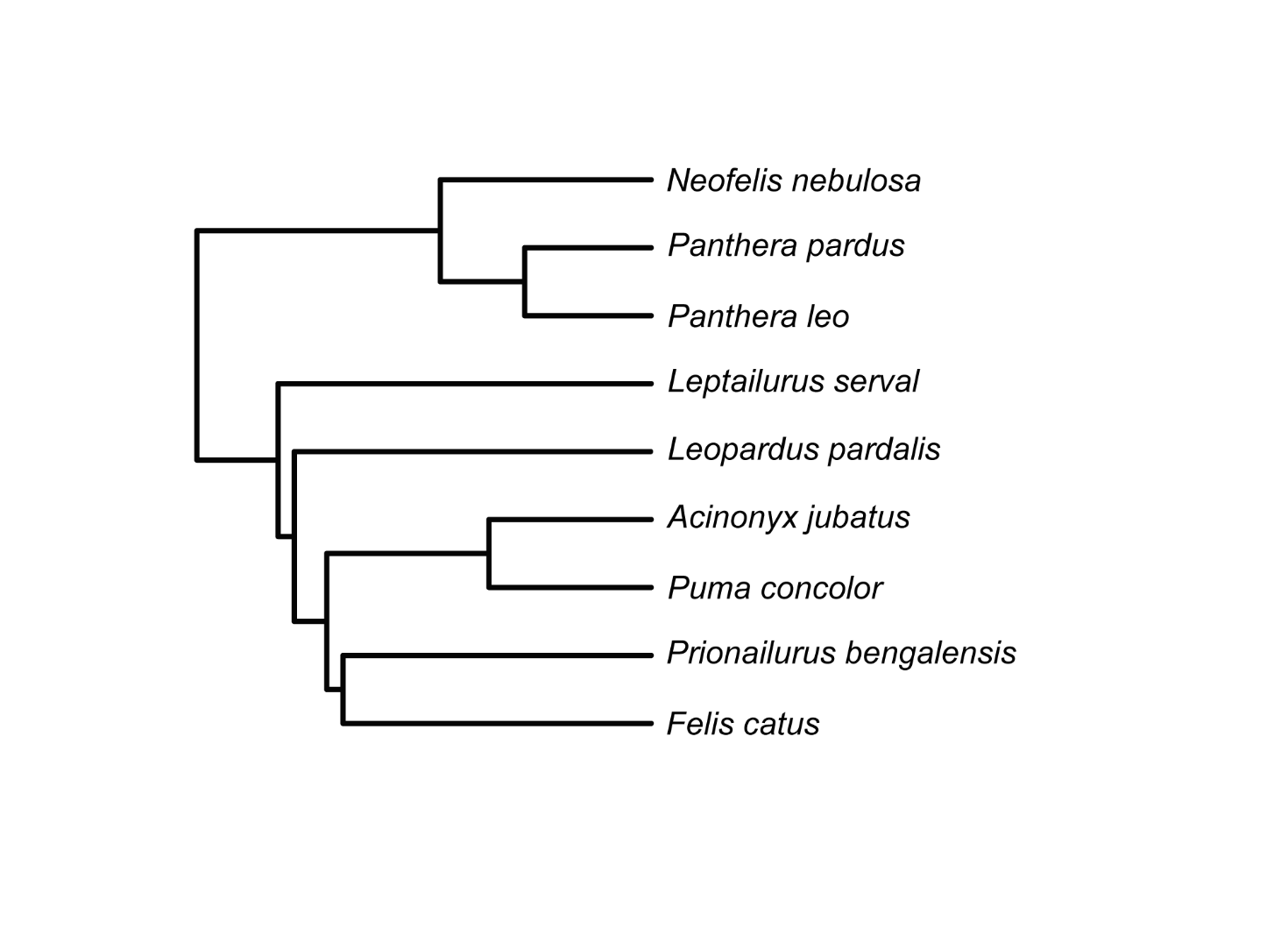

Supplement: Supplementary file 1 — Supplementary material 1 (DOCX 204 KB) [file 11692_2017_9443_MOESM1_ESM.docx]
